# Evaluating transcriptomic integration for cyanobacterial constraint-based metabolic modelling

**DOI:** 10.3389/fbinf.2026.1715377

**Published:** 2026-02-04

**Authors:** Thomas Pugsley, Guy Hanke, Christopher D. P. Duffy

**Affiliations:** 1 School of Biological and Behavioural Sciences, Queen Mary University of London, London, United Kingdom; 2 Digital Environment Research Institute, Queen Mary University of London, London, United Kingdom

**Keywords:** Flux Balance Analysis, autotrophic flux distributions, cellular phenotypes, central carbon metabolism, constraint-based metabolic models, cyanobacteria, validation

## Abstract

Metabolic modelling has wide-ranging applications, including for the improved production of high-value compounds, understanding complex diseases and analysing microbial community interactions. Integrating transcriptomic data with genome-scale metabolic models is crucial for deepening our understanding of complex biological systems, as it enables the development of models tailored to specific conditions, such as particular tissues, environments, or experimental setups. Relatively little attention has been given to the validation and comparison of such integration methods in predicting intracellular fluxes. While a few validation studies offer some insights, their scope remains limited, particularly for organisms like cyanobacteria, for which little metabolic flux data are available. Cyanobacteria hold significant biotechnological potential due to their ability to synthesise a wide range of high-value compounds with minimal resource inputs. Using existing transcriptomic data, we evaluated different methodological options that can be taken when integrating transcriptomics with a genome-scale metabolic model of *Synechocystis* sp. PCC 6803 (iSynCJ816), when predicting autotrophic flux distributions. We find METRADE* (using single objective optimisation) to be the best-performing method in cyanobacteria owing to its ability to perform well across both metrics but emphasise the importance of configuration and scaling in achieving these outcomes.

## 1 Introduction

Metabolic models are useful tools for the prediction of metabolic fluxes and cellular phenotypes, with wide ranging applications across biotechnology (Yasemi and Jolicoeur, 2021). Constraint-based metabolic models are mathematical representations of cells which enable system-level analyses of metabolism. These networks, where reactions are modelled as edges and metabolites as nodes, encompass important biological system properties. Flux is allocated in a manner which obeys reaction stoichiometry with the overarching reaction topology facilitating the role of interconnectedness within the network. Gene-protein interactions are represented as Boolean expressions, useful when determining gene essentiality and often used when integrating external data (like in the case of transcriptional integration). Finally, network constraints influence and impose limits on flux capacity throughout the network. Thermodynamic constraints set reaction directionality, and nutrient availability modelling indirectly constrains the model. Often, constraints are applied on nutrient exchange reactions to reflect experimental conditions, making metabolic models highly useful tools for modelling adaption to varying environments. In the case of transcriptional integration, additional constraints are applied to the model to redirect flux predictions (Joshi et al., 2017; Smith et al., 2013; Machado and Herrgård, 2014; Young et al., 2011; Lea-Smith et al., 2021).

Typically, metabolic models are solved when flux is allocated throughout the network in order to maximise an objective (typically, a biomass pseudo-reaction). It is through this systems-level optimisation that metabolic models can predict cellular behaviour such as growth rates, metabolite secretion and gene/pathway modifications. It is this linking of the gene and reaction data (used to develop GEMs) to phenotypic predictions which make metabolic models powerful tools in biotechnology: identifying gene targets for high-value compound production or elucidating pathways of interest involved metabolic reprogramming in changing conditions for *in vitro* study. A comparative analysis for integrating transcriptomic data into metabolic models for cyanobacteria, would therefore be useful to researchers seeking to optimise and understand metabolism in photosynthetic bacteria.

Covert et al. (2001) published the first method to integrate transcriptomic data with metabolic models, enabling model simulations to account for gene regulation (Covert et al., 2001). In the following decades, many more integration methods were developed, allowing researchers to capture condition-specific properties of metabolic flux distributions, enhancing the specificity of downstream analyses (Bordbar et al., 2012; Zur et al., 2010; Caroline et al., 2009; Angione and Lió, 2015; Agren et al., 2014).

Integration methods can be broadly divided into two categories: switch-based and valve-based (Hyduke et al., 2013; Vijayakumar et al., 2018). Switch-based methods use thresholding to categorise reactions based on their predicted activity, with reactions of low predicted flux typically being switched off or mathematically penalised for carrying flux. This type of integration has predominantly been used to model human cell types due to its binary on/off strategy, with previous studies having examined the impact of specific methodological decisions during integration (Richelle et al., 2019; Gopalakrishnan et al., 2023; Joshi et al., 2020). Richelle et al. (2019) examined gene mapping types, thresholding and the order of those steps for the creation of human tissue-specific models while Gopalakrishnan et al. (2023) mainly focused on algorithmic details and changing thresholding cut-offs in *E. coli*, Chinese Hamster Ovary (CHO) and a renal cancer cell line. Joshi et al. (2020) also examined transcriptomic integration with a cancer cell line model, focused mainly on the influence of thresholding values. iMAT is a popular switch-based integration method with its implementations being used to further understand metabolic function in human tissues, stem cell metabolism and in identifying epigenetic dependencies to drug response (Shlomi et al., 2008; Lin et al., 2025; Shen et al., 2019b; Shen et al., 2019a).

Valve-based methods involve modifying reaction bounds in a continuous manner, with bounds being relaxed for upregulated reactions and tightly constrained for downregulated reactions. Although enzyme activity is not always directly correlated with transcript levels, these methods assume that gene expression can be used as a “soft” constraint to approximate an upper bound for reaction rates. It has been argued that valve-based approaches are preferable as they do not suffer from the loss of fine-grained expression changes in the same way as switch-based methods (Machado and Herrgård, 2014). METRADE (Angione and Lió, 2015) and E-flux2 (Kim et al., 2016) [originally E-flux (Caroline et al., 2009)] are examples of valve-based integration methods, both of which have been applied to microbial metabolic models. In the case of the original E-flux, Caroline et al. (2009) used their newly developed integration method to model mycolic acid biosynthesis, predicting drug responses. Since then, it has become a popular integration method and has been followed by the development of a second version, E-flux2 (Kim et al., 2016). E-flux2 ensures flux predictions incorporate minimisation of the Euclidean norm alongside maximisation of the objective ensuring unique solutions (Kim et al., 2016). METRADE (MEtabolic and TRanscriptomics ADaptation Estimator), on the other hand, developed by Angione and Lió (2015), has been implemented as a multi-objective optimisation problem. Originally utilised for the creation of multi-omic models of *E. coli*, METRADE has since been applied to the cyanobacterial species *Synechococcus* sp. PCC 7002. Using a hybrid machine learning and metabolic modelling pipeline, repsonse mechanisms to light and salinity fluctuations were detected - a finding which was not possible from the analysis of the transcriptomic data alone (Vijayakumar et al., 2020).

13C metabolic flux analysis (13C-MFA), in which cells are fed 13C-labelled substrates and enrichment patterns calculated, is widely accepted as the gold-standard for quantifying flux through central carbon metabolism (Wiechert, 2001; Zamboni et al., 2009; Beurton-Aimar et al., 2011). For this reason, 13C-MFA measurements are typically used to validate flux predictions by metabolic models and the use of integration methods, as they provide systems-wide datasets for comparison (Nielsen, 2003). However, 13C-MFA experiments are notoriously challenging to perform and costly (Kim et al., 2016). For this reason, few extensive 13C-MFA datasets exist (relative to transcriptomic studies), even for well-studied organisms such as *E. coli* and *S. cerevisiae* (Guo et al., 2015). Unsurprisingly, there is even greater dataset scarcity among cyanobacteria, with only 3 central carbon flux distributions having ever been published alongside paired transcriptomics (Bhadra-Lobo et al., 2020; You et al., 2015; You et al., 2014; Young et al., 2011). Methods for assessing flux predictions from context-specific models in cyanobacteria are therefore severely limited. Complicating matters further, cyanobacterial systems present distinct challenges compared to their heterotrophic counterparts. In particular, the validity of inferring photosynthetic fluxes from transcript profiles has yet to be robustly assessed, and the role of model lighting configurations, scaling strategies and optimal threshold combinations remain relatively unexplored.

**FIGURE 1:**
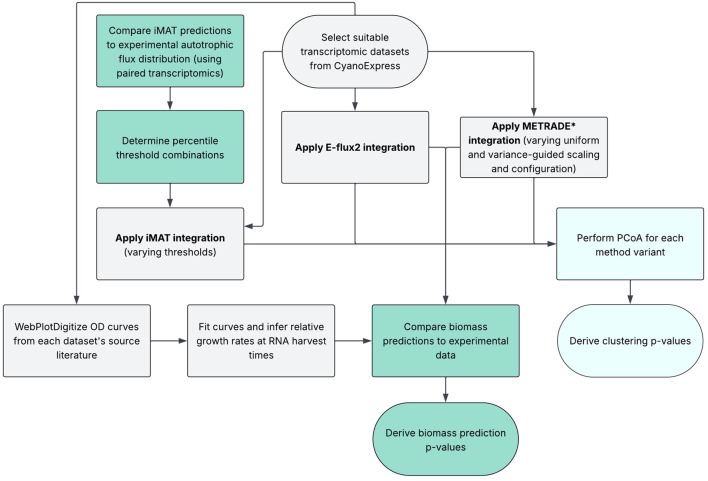
An overview of the analysis pipeline: To derive a dataset of measurable traits, paired OD plots from each dataset’s respective source literature were used to infer relative changes in growth rates at the time of RNA harvest. This experimental data was compared to growth rate predictions by E-flux2 and METRADE* and p-values calculated. In the case of iMAT, which does not explicitly predict biomass rates, we used the only published autotrophic MFA dataset (Young et al., 2011) to determine which threshold combinations resulted in biologically reasonable flux predictions (Supplementary Figure S1). Only highly performing combinations (based on $R^{2}$ and RMSE metrics) were carried forward for use in PCoA analysis alongside METRADE* and E-flux2 implementations.

In this study, we present a novel pipeline (Figure 1) to evaluate the performance of integration methods for the creation of context-specific models of *Synechocystis* sp. PCC 6803 (hereon referred to as *Synechocystis*) using existing expression profiles in CyanoExpress (Hernandez-Prieto and Futschik, 2012). We selected 7 time-series datasets harvested from WT and mutant cells grown in varying conditions for analysis. All sets of flux predictions could then be assessed for their ability to capture condition-specific properties of the data using Principle Coordinates Analysis (PCoA). This was to determine how well context-specific models representing specific conditions clustered together (Gower, 1966). For robust validation in the absence of comprehensive MFA data, we determined that an opposing metric was required to validate a separate property of the system predictions. Here, we propose a dual-metric set-up, where condition-specificity of full flux distributions is considered alongside predictions of a continuous measurable trait to confirm successful contextualisation.

## 2 Methods

### 2.1 Optimisation

Using the COBRA Toolbox version 3 (Laurent et al., 2019) for MATLAB, Flux Balance Analysis (FBA) was used to predict growth rates at different time points using the *Synechocystis* metabolic model, iSynCJ816 (Joshi et al., 2017).

In metabolic modelling, the metabolic network is represented as a stoichiometric matrix S , where each element $S_{ij}$ corresponds to the stoichiometric coefficient of metabolite i in reaction j . The matrix S has dimensions m×n , where m is the number of metabolites and n is the number of reactions.

$$S=\left(\begin{array}{cccc} S_{11} & S_{12} & \cdots & S_{1n}\\ S_{21} & S_{22} & \cdots & S_{2n}\\ \vdots & \vdots & \ddots & \vdots \\ S_{m1} & S_{m2} & \cdots & S_{mn} \end{array}\right)$$

The system is assumed to be at steady-state, resulting in this system of linear equations:

S⋅v=0

where v is the vector of reaction fluxes. Each element $v_{j}$ in v represents the flux through reaction j .

In all model setups, to simulate autotrophic growth, glucose import was switched off and bicarbonate import was modelled as the sole source of carbon to the system.

### 2.2 Integration methods

Key features of each integration method are shown in Table 1.

**TABLE 1:** Key features of integration methods.

Name	Mapping function	Thresholding	Scaling	Configuration	Biomass prediction
METRADE*	Lazy-step	No	One-size-fits-all	Yes	Yes
METRADE* importance	Lazy-step	No	Reaction-specific	Yes	Yes
E-flux2	Linear	No	No	No	Yes
iMAT	No	Yes	No	No	No

### 2.3 METRADE*

For all METRADE*- (and E-flux2-based) simulations, the objective function was set to the autotrophic biomass equation.

$$\text{maximize}\;Z=c^{T}\cdot v$$

where c is a (transposed) vector that specifies the objective function and Z is the scalar result of the optimisation. Reaction fluxes are subject to upper and lower bound constraints. Ultimately, these are determined by the integration method.

Once reaction expressions have been derived for all reactions, they can be converted into new reaction bounds using a mapping function:

$$v_{\min}\;\phi \left(\Theta \right)\leq v_{i}\leq v_{\max}\;\phi \left(\Theta \right)\;$$

METRADE* uses the lazy-step mapping function (Angione and Lió, 2015):

$$\phi \left(\Theta \right)={\left[1+\gamma \left|\log\left(\Theta \right)\right|\right]}^{sgn\left(\Theta -1\right)}$$ (1)

where Θ is the computed reaction expression and γ is a scaling parameter. We tested the same γ ranges as those used by Vijayakumar et al. (2020) where they applied METRADE to a cyanobacterial model (Vijayakumar et al., 2020).

In order to best predict realistic flux distributions, we modified METRADE’s original multi-objective formulation from Angione and Lió (2015) to maximise a single biomass objective. To ensure unique solutions and maximise biological plausibility, L1 regularisation was ensured by implementing Parsimonious Flux Balance Analysis (pFBA) - differing from the L2 regularisation approach of E-flux2 (Kim et al., 2016). The use of a two-stage linear program to find solutions maximising biomass rates while simultaneously minimizing total metabolic flux is routed in the idea that cells avoid energetically expensive means for achieving optimal production. We refer to our modified single objective of METRADE as METRADE* to distinguish it from its original implementation (Angione and Lió, 2015):

1. Solve for maximal biomass:

$$\text{maximize}\;Z=c^{T}\cdot v$$

2. Minimize total flux subject to optimal growth:

$$\begin{array}{cc} \text{minimize}\; & \underset{i}{\sum }|v_{i}|\\ & S\cdot v=0\\ & v_{\min}\cdot \phi \left(\Theta \right)\leq v\leq v_{\max}\cdot \phi \left(\Theta \right) \end{array}$$

The above constraints were applied to both steps, and pFBA was solved using the Gurobi solver (Gurobi Optimization and LLC (2024).

#### 2.3.1 METRADE* configurations

In the context of this study, configurations refer to the initial bound setup of the metabolic model prior to bound alteration by an integration method. We tested integration methods starting with two different model configurations. For both configurations, all non-zero bounded reactions in the default model were set to an arbitrary value of 1000 or -1000. In configuration “Baseline”, allowable flux through the photon input reaction was set to half of that of the rest of the system bounds (−500). The purpose of this was to create a buffering effect so that the supply of light to the wider system was less likely to be influenced by transcriptional changes associated with the photosystems. For the other configuration, “Max”, photon import was capped at the same upper bound as the rest of the system (−1000). All bound units are mmol/gram dry cell weight/hour.

#### 2.3.2 METRADE* scaling

For METRADE*-based methods, we used two scaling strategies: “one-size-fits-all”’ (in which γ in Equation 1 was scaled to the same value across all reactions) and “reaction-specific” - also referred to as “importance-based” scaling: We define the importance of a gene in the same way as Angione and Lió (2015). The scaling parameters tested, in the context of importance, relate to the maximum gene importance value. For each gene in each condition, gene variance values were computed from the expression profiles. The maximum gene importance value was used to normalise these variances such that

$$\text{gene importance}=\text{max gene importance}\times \frac{1}{\sigma_{i}/\text{min var.}}$$

where $\sigma_{i}$ is the variance of gene i and min var. is the minimum variance value of the variances dataset (Angione and Lió, 2015). For each gene in the model, we determined whether it was present in the expression profiles. Letting G be the set of genes in the model, and D be the set of genes in the transcriptomic dataset, gene importance $\gamma_{i}$ is defined as:

$$\gamma_{i}=\left\{\begin{array}{cc} \frac{\text{max gene importance}}{2}\; & \text{if }G_{i}\notin D\\ \text{gene importance}\left(G_{i}\right)\; & \text{if }G_{i}\in D \end{array}\right.$$

This approach aims to emphasise the constraint-based influence of variably expressed genes while dampening bound changes from stably expressed genes. Missing genes are neither amplified nor dampened to reduce their influence on the solution space.

### 2.4 E-flux2

In E-flux2 (Kim et al., 2016), reaction upper bounds $\left(v_{\max}\right)$ are set to the corresponding reaction expression values θ . For reversible reactions, the lower bounds $\left(v_{\min}\right)$ are set to −θ , and for irreversible reactions, they remain at zero. FBA is run using the modified model to find a feasible flux distribution $v_{0}$ that maximizes the biomass objective. (While there is no mapping function defined, the fixing of reaction expressions as bound constraints in E-flux2 is equivalent to the use of a linear mapping function.)

1. Solve for maximal biomass:

$$\text{maximize}\;Z=c^{T}\cdot v$$

2. A convex quadratic programming (QP) problem is solved:

$$\begin{array}{cc} \text{minimize} & \underset{i}{\sum }{\left|v_{i}\right|}^{2}\\ \text{subject to}\; & S\cdot v=0\\ & c^{⊤}\cdot v=c^{⊤}\cdot v_{0},\\ & v_{\min}\leq v\leq v_{\max} \end{array}$$

The above constraints were applied to both steps, and the optimisation was solved using the Gurobi solver (Gurobi Optimization and LLC (2024).

### 2.5 iMAT

In the case of iMAT (Zur et al., 2010), gene expression data are discretised into three categories: highly, low and neutral expression. The assignment of reactions to each of these categories depends upon user-defined upper and lower threshold values. Highly and lowly expressed reactions are linked to binary variables in the problem formulation which indicate whether reaction flux is consistent with its expression state category. iMAT integration therefore seeks to find a flux distribution which is maximially consistent with the input gene expression data, through these reaction state categories.

Highly expressed (H) : expression ≥ upper threshold.

Lowly expressed (L) : expression between 0 and lower threshold.

Ambiguous (A) : all other reactions (not constrained)

The binary variables $y_{j}\in \left\{0,1\right\}$ for each reaction in H∪L indicate whether the reaction is consistent with its expression state classification. The objective is to maximize the number of consistent reactions:

$$\max\underset{j\in H\cup L}{\sum }y_{j}$$

Subject to the following constraints:

$$\begin{array}{cccc} & S\cdot v=0 & & \left(\text{steady}-\text{state}\right)\\ & v_{\min}\leq v\leq v_{\max} & & \left(\text{original bounds}\right)\\ & v_{j}\geq \varepsilon \cdot y_{j}\; & & \forall j\in H\\ & v_{j}\leq v_{\max,j}+\epsilon \cdot \left(1-y_{j}\right)\; & & \forall j\in H\\ & v_{j}\leq \epsilon \cdot \left(1-y_{j}\right)\; & & \forall j\in L\\ & v_{j}\geq v_{\min,j}-\epsilon \cdot \left(1-y_{j}\right)\; & & \forall j\in L\\ & y_{j}\in \left\{0,1\right\}\; & & \forall j\in H\cup L \end{array}$$

ϵ is typically a small positive value (here, ϵ=1 ), and ε is a small threshold (here, 1e−8 ) to define nonzero flux for high-expression reactions. Reactions in A (ambiguous) are unconstrained beyond their default.

The optimization problem is formulated as a mixed-integer linear program (MILP) and solved using the Gurobi solver (Gurobi Optimization and LLC, 2024).

### 2.6 Transcriptomic data

Expression profiles deposited on CyanoExpress 2.3 (pre-processing and normalisation details available from: http://cyanoexpress.sysbiolab.eu/) were screened for their suitability for downstream analysis. Each transcriptomic dataset was required to have expression profiles from a minimum of 3 independent timepoints and have paired OD data to infer growth rates from (within their associated source papers). WebPlotDigitizer was used to extract experimental data (Supplementary Figure S2) from published OD plots (Rohatgi, 2011). Transcriptomic log-fold change values were converted to fold-change (so that wild-type expression was equal to 1) in all cases (except to determine optimal threshold combinations for iMAT) before use for metabolic modelling.

In the case of determining which iMAT threshold combinations were carried forward to be applied to the CyanoExpress datasets, raw normalised read counts were used as input (Young et al., 2011). Since iMAT relies on percentile-based discretisation of the input data, outputs and optimal thresholds are equivalent to those which are applied to fold-change transcript data. Gene mapping to the metabolic model required conversion of accessions using the ASM972v1 genome assembly: https://www.ncbi.nlm.nih.gov/datasets/genome/GCF_000009725.1/.

### 2.7 Growth rate derivation

**FIGURE 2:**
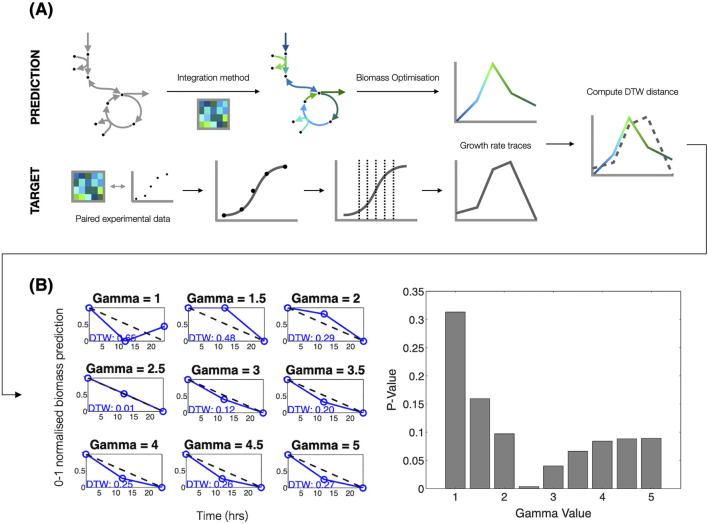
Growth rate methodology schematics **(A)** showing how optical density data associated with transcriptomic measurements at multiple timepoints were fit with curves, allowing for growth rate estimation in cases where authors did not measure OD at the time of RNA harvest. Curve fitting thereby extended the number of viable datasets suitable for downstream analysis. Integration methods were used to contextualise the metabolic model using the expression profiles from each timepoint. Maximising the biomass objective function was used to generate growth predictions at each time point, facilitating comparison of the predicted and experimentally-derived growth rate traces by computing DTW distances. **(A)** Snapshot of the biomass prediction pipeline. **(B)** (METRADE* using uniform scaling applied to the crhR dataset) is shown. Scaling parameter changes (left panel of B) influence the relative differences between time series biomass predictions. The right panel of **(B)** shows the resulting p-values after comparing observed DTW values (in blue; left panel) to the null distribution.

Since absolute growth rate determination is not possible from optical density data alone, we derived estimates for relative growth rates. These relative growth rate traces were derived by inferring values from fitted OD curves (Figure 2A). For each OD plot, either an exponential, exponential rise, or logistic curve. The fitted curve’s parameters were used to estimate the growth rate as outlined below.

The optical density is defined as,

$$OD\left(t\right)=-\log_{10}\left(T\left(t\right)\right)$$

where T(t) is the transmittance as a function of time. In the limit of an infinitely far detector,

$$T\left(t\right)=\exp\left(-\sigma lc\left(t\right)\right)$$

where σ is the scattering cross-section of the particles/cells, l is the optical path, and c(t) is the time-dependent concentration. If we take the derivative of OD(t) ,

$$\frac{dOD}{dt}=\frac{dc}{dt}\cdot \left(\frac{\partial OD}{\partial c}\right)=\frac{dc}{dt}\cdot \frac{\sigma l}{\ln\left(10\right)}$$

Therefore, the growth rate is proportional to the rate of change of optical density,

$$\frac{dOD}{dt}\propto \frac{dc}{dt}$$

If we normalise $\frac{dOD}{dt}$ to its maximum rate,

$$r\left(t\right)=\frac{\frac{dOD}{dt}}{\max\left(\frac{dOD}{dt}\right)}$$

Then this provides a measure of the relative growth rate,

$$r\left(t\right)\in \left[0,1\right]$$

Generally, OD(t) is measured at discrete time points, so we must fit simple functions to the data.

#### 2.7.1 Exponential growth

If the experiment only measures growth in the initial exponential phase, then the growth curve will follow:

$$OD\left(t\right)=ae^{kt}$$

where a is a constant (starting OD) and k is the rate constant. Therefore, the growth rate is

$$\frac{dOD}{dt}=ake^{kt}=kOD\left(t\right)$$

So, the relative growth rate is

$$r\left(t\right)=\frac{kOD\left(t\right)}{\max\left(kOD\left(t\right)\right)}=\frac{OD\left(t\right)}{\max\left(OD\left(t\right)\right)}$$

#### 2.7.2 Saturating growth

If the measurement only captures the linear phase followed by saturation, then the OD curve should fit

$$OD\left(t\right)={OD}_{\max}\left(1-e^{-kt}\right)$$

where ${OD}_{\max}$ is the maximum optical density. Taking the derivative:

$$\frac{dOD}{dt}={OD}_{\max}ke^{-kt}$$

and normalising to the maximum rate

$$r\left(t\right)=\frac{{OD}_{\max}ke^{-kt}}{\max\left({OD}_{\max}ke^{-kt}\right)}=e^{-kt}$$

#### 2.7.3 Logistic growth

If the measurement captures full logistic growth, then the OD curve fits:

$$OD\left(t\right)=\frac{{OD}_{\max}}{1+e^{-k\left(t-t_{0}\right)}}$$

where k is the logistic growth constant and $t_{0}$ is the time at which OD reaches half of its maximum:

$$OD\left(t_{0}\right)=\frac{{OD}_{\max}}{2}$$

Taking the derivative:

$$\frac{dOD}{dt}={OD}_{\max}\cdot \frac{ke^{-k\left(t-t_{0}\right)}}{{\left(1+e^{-k\left(t-t_{0}\right)}\right)}^{2}}$$

Normalising to the maximum rate:

$$r\left(t\right)=\frac{\frac{dOD}{dt}}{\max\left(\frac{dOD}{dt}\right)}=\frac{4e^{-k\left(t-t_{0}\right)}}{{\left(1+e^{-k\left(t-t_{0}\right)}\right)}^{2}}$$

### 2.8 Assessment of predictions

#### 2.8.1 Data normalisation

To allow meaningful comparisons between predicted and experimentally-derived growth rate traces, both datasets were scaled to a 0–1 range (Figure 2A). Given a set of reference data $y_{1}=\left\{y_{1}^{\left(1\right)},y_{1}^{\left(2\right)},\ldots ,y_{1}^{\left(n\right)}\right\}$ and prediction data $y_{2}=\left\{y_{2}^{\left(1\right)},y_{2}^{\left(2\right)},\ldots ,y_{2}^{\left(m\right)}\right\}$ , min-max normalisation for each data point $y_{i}^{\left(j\right)}$ was performed using:

$$y_{\text{norm}}^{\left(j\right)}=\frac{y^{\left(j\right)}-y_{\min}}{y_{\max}-y_{\min}}$$

where $y_{\min}$ and $y_{\max}$ are the minimum and maximum values of the dataset, respectively.

#### 2.8.2 Dynamic time warping

DTW was used to assess temporal growth rate trace similarity. Given a reference $y_{1}$ and prediction trace $y_{2}$ , for each pair of points (i,j) , the squared difference (cost) between the elements of the sequences was computed as follows:

$$\text{cost}\left(i,j\right)={\left(y_{1}^{\left(i\right)}-y_{2}^{\left(j\right)}\right)}^{2}$$

The DTW matrix was populated using the following recurrence relation:

$$\begin{array}{cc} DTW\left(i,j\right) & =\text{cost}\left(i,j\right)+\min\left(DTW\left(i-1,j\right),\right.\\ & \;\left.DTW\left(i,j-1\right),DTW\left(i-1,j-1\right)\right) \end{array}$$

where i∈{1,2,…,n} and j∈{1,2,…,m} . The final DTW distance between the two traces is then given by:

$$\text{DTW distance}=\sqrt{DTW\left(n,m\right)}$$

The final distance gives the optimal alignment that minimises the cumulative distance between both growth rate traces.

#### 2.8.3 P-value derivation

For each set of predictions from a particular integration method, condition-specific growth traces were individually normalised to facilitate comparison using DTW. For each condition, random growth rates were computed for each time-point in the condition dataset, with the resulting growth trace max-min normalised. For this normalised growth trace, a DTW distance was computed against the relevant condition’s experimentally derived growth trace. This process of randomly generating DTW distances was repeated for each condition 100,000 times (determined using a convergence analysis) to form condition-specific null distributions. P-values were then computed as the proportion of randomly generated DTW distances lower than an integration method’s prediction (Figure 2B).

### 2.9 Condition discrimination

#### 2.9.1 Principal coordinates analysis (PCoA)

To visualise structural similarities between predicted flux distributions across different conditions, PCoA was performed. To ensure consistency between all flux vector dimensions between methods (prior to pre-processing) and to remove negative flux values, all reversible reactions were modelled as separate forward and backward reactions (as performed by pFBA by default). For each integration method, predicted fluxes below solver tolerance $\left(<10^{-10}\right)$ were set to zero, and reactions with zero flux across all conditions were excluded. Column-wise total normalisation was performed (with each set of flux distributions summing to 1) before the removal of low variance reactions (standard deviation $<10^{-3}$ ) and reaction fluxes were z-score normalised across reactions. We produced a projection for every integration method individually, each with flux predictions from all conditions. Pairwise Euclidean distances were computed between all condition vectors to generate a dissimilarity matrix and classical multidimensional scaling (MDS) was applied to this matrix using the scikit-learn implementation; mathematically equivalent to PCoA. The first two principal coordinates were retained, and resulting 2D embeddings used for visualisation. The proportion of variance explained was estimated from the variance of the MDS embedding along each dimension and normalising by the total variance.

#### 2.9.2 P-value derivation

**FIGURE 3:**
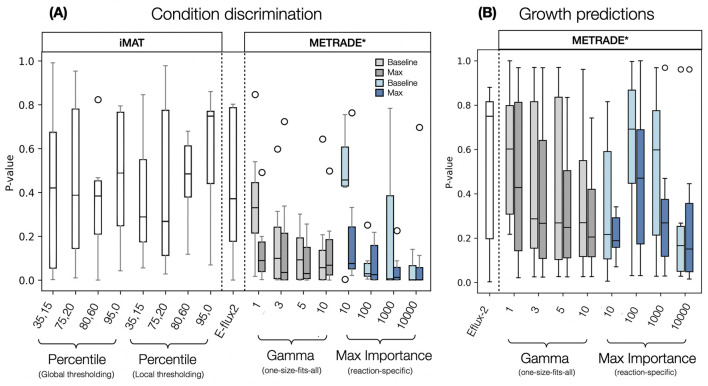
Distribution of p-values obtained for all integration method implementations. Condition discrimination p-values represent the ability of an integration method to form condition-specific clusters in low dimensionality space with all 7 condition datasets **(A)**. Growth p-values indicate the ability of an integration method to match estimated relative growth rate traces for all conditions **(B)**. In the case of iMAT, x-axis labels refer to upper and lower percentile input thresholds. For METRADE*, one-size-fits-all scaling values are uniform gamma values while x labels for the reaction-specific METRADE* is maximum importance. [Boxes represent 25th and 75th percentiles with median values indicated in the centre. Whiskers show smallest and largest values within 1.5 IQR and circles are outliers. Figure made using Matplotlib (Hunter, 2007)].

We performed permutation tests comparing intra-condition mean Euclidean distances to a null distribution to quantify each integration method’s ability to capture condition-specific properties. For each condition group (Table 2) within a given integration method’s set of predictions, we computed the mean pairwise Euclidean distance between samples in the projection belonging to the same condition set. Using all samples in the projection, we randomly sampled groups of the same size (without replacement) and computed their average pairwise distances for 100,000 iterations (determined using a convergence analysis). A p-value for a particular condition was calculated as the proportion of random permutation-derived mean Euclidean distances lower than that observed from the actual condition set. For each integration method, we performed this analysis for each of the 7 condition sets to yield a distribution of p-values, plotted in Figure 3A. (An example is provided on GitHub: https://github.com/ThomasPugsley/Evaluating_transcriptomic_integration and a schematic is available: Supplementary Figure S3.).

**TABLE 2:** Expression profile labels and their source publications - more details available from CyanoExpress (Hernandez-Prieto and Futschik, 2012).

Plotting name	Description	Sample size	References
Cd	Cadmium stress	9	Houot et al. (2007)
Blue light	Blue light growth	6	Singh et al. (2009)
High light	High light stress	6	Singh et al. (2008)
CrhR	CrhR-mutant; Low temperature	3	Prakash et al. (2010)
S starvation (H)	Sulphur starvation (no HEPES)	3	Zhang et al. (2008)
S starvation	Sulphur starvation	7	Zhang et al. (2008)
Iron stress	Iron stress	5	Hernández-Prieto et al. (2012)

## 3 Results and discussion

After screening the datasets in CyanoExpress, 7 sets of expression proiles were deemed suitable for downstream analysis (Table 2). We implemented our novel dual-metric pipelines using the 7 transcriptomic datasets (Figure 1).

PCoA clustering revealed marked differences in performance metrics across methods used for condition discrimination (Figure 3). Of the methods tested, iMAT’s performance appeared most heavily influenced by input parameter choices. The more unusual 35th and 15th Local percentile thresholding combination resulted in surprisingly tight condition clusters, outcompeting Eflux-2 and with a median roughly on par with the more typical iMAT implementation using 75th and 20th percentile thresholds. METRADE*, in contrast, demonstrated a reasonable level of robustness across scaling parameters and scaling strategies, particularly for predictions using gamma values greater than 1, and maximum importance values greater than 10. E-flux2, which does not require any user-specified input parameters, performed moderately well with a median p-value of ∼0.4 (Figure 3A). The majority of METRADE* implementations, however, outcompeted E-flux2. Among the METRADE* implementations tested, variance-based scaling, using maximum importance values of 100, 1000 (in the case of the max configuration) and 10,000, resulted in the tightest condition clusters. This is unsurprising since the scaling strategy ensures that genes with greatly altered expression across the time-series, have their bounds modified to a greater extent than those which are invariant. Importantly, uniformly-scaled METRADE* also performed well, and had greater robustness across scaling parameter choices.

METRADE* configuration changes seemed to have minimal impact on the capacity of the model to discriminate between conditions in PCoA space. While uniformly-scaled METRADE* seemed to favour the Max configuration for the majority of implementations, the dependency seems to switch at gamma = 10. METRADE*’s apparent robustness to changes in configuration is surprising as the Baseline setup creates buffering, forcing the reshaped solution space to exert more influence deeper within the reaction network, rather than causing more localised changes in flux utilisation, for example, around photosynthesis - the point of entry for flux to the system. It is unsurprising that for low gamma values, the Max configuration was clearly favoured since buffering creates a larger gap between the new bounds and allowable flux, meaning a “stronger” mapping is required for contextualisation to take effect.

The initial configuration of the model has the potential to alter predictive accuracy as the treatment of the model’s Boolean gene-reaction rules assume reaction fluxes are controlled by enzymes. Cyanobacterial photosystems, which are not themselves regulated like enzymes, are represented in iSynCJ816’s photosystem reactions, and are the first point at which transcriptional integration can influence light entering the wider system. It can therefore be argued that these “points of entry” for light should not be subject to strict bound changes based on expression profiles, due to an inappropriate assumption that they are regulated in the same manner as enzymatic reactions. To illustrate a violation of this assumption, a typical high light stress response can be considered. It would be expected that as a long-term strategy, photosystem proteins would be downregulated to prevent oxidative damage, as excitation input would exceed electron sink capacity of downstream of downstream photosynthetic pathways. Without specifying explicit estimates for photon input in the metabolic model, after integration with transcriptomic data, such a response at the genetic level would result in a reduction of potential flux entering the system due to the shrinking of flux bounds at the photosystems. Such an assumption is inappropriate given the numerous non-transcriptional regulatory mechanisms which act to maintain flux through photosystem reactions (Uzuner Odongo et al., 2025; Shen et al., 2019a; Beurton-Aimar et al., 2011). Energy redistribution through phycobiliosome state transitions and dissipation of excess energy by non-photochemical quenching are key examples of this, both of which act with near immediacy (Joshi et al., 2017; Shlomi et al., 2008). Therefore, in our analyses, the “Baseline” configuration (for METRADE* analyses) was set up so that the upper bounds of the photosystem reactions (and all other reactions in the system) were twice as large as the available light, creating a buffer between any transcription-induced bound modification and light entering the system. In the “Max” configuration, photosystem upper bounds were set to equal the available light, meaning any modification of the photosystem bounds could reduce light entering the system based on the gene-reaction rules.

**FIGURE 4:**
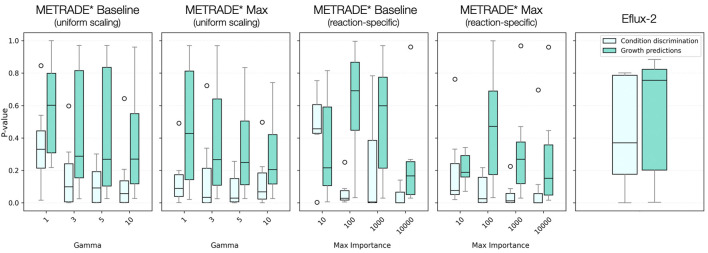
Distribution of p-values for condition discrimination offset against growth rate predictions. Different methods are indicated above graphs - E-flux2 is included as a benchmark. Colours reflect the two types of metric, consistent with coloured labelling in Figure 1. [Boxes represent 25th and 75th percentiles with median values indicated in the centre. Whiskers show smallest and largest values within 1.5 IQR and circles are outliers. Figure made using Matplotlib (Hunter, 2007)].

It may be expected that Baseline versions of METRADE* would perform better when capitalising upon light input buffering as they would be less susceptible to severe bound changes near the photosystems. However, for simulations with adequately large scaling values, there appeared to be no clear difference in performance using each configuration. The Max configuration, more expectedly, was preferred when predicting growth rates across the majority of implementations (Figure 3B). This is likely due to flux inference at photosynthesis being closely tied to overall productivity and therefore biomass rates. Since the Max configuration encourages solution space reshaping around photosynthesis, it appears the most reliable configuration for predicting growth rates, despite the assumptions outlined above. It is important to acknowledge that while biomass predictions are useful for gauging proportionate transcriptional integration ‘strength’, some metabolite secretion rates may not benefit so dramatically from using the Max configuration if they are determined by solution reshaping deeper within the network. Both performance metrics suggest that use of uniform scaling with METRADE* can be suitable for successful contextualisation but agree that gamma values need to be sufficiently high to accurately model cellular metabolism (Figures 3A,B). Using dual-metric visualisation, it becomes evident that some input setups allow for successful condition discrimination but remain unable to predict growth rates with good accuracy (Figure 4). When using uniform scaling, METRADE* performed better when using higher scaling parameters, with the best predictions at gamma = 10 (using the Max configuration). There was substantial variability in predictions when using importance-based scaling. Predictions using maximum importance values of 10,000 were, however, the best across all methods tested (only marginally in the case of Max) and broadly consistent across both configurations (Figure 4).

Autotrophic constraint-based systems present unique challenges for contextualisation. The dominance of light input as an essential energy source for autotrophs means the single photon exchange reaction has a disproportionately high uptake rate compared to other essential model reactions such as bicarbonate and water exchange (Joshi et al., 2017). Transcriptomic integration with heterotrophic systems is less susceptible to the biases introduced by such a ‘top-heavy’ setup as they rely on multiple essential exchange reactions like carbohydrate, oxygen and nitrogen import - all carrying flux at rates typically within an order of magnitude of each other (Van ‘t Hof et al., 2022; Blázquez et al., 2023). For methods like METRADE, where all default “unconstrained” reaction bounds (prior to integration) are set to the same magnitude, exchange reactions and their associated downstream pathways which support high flux rates, will be more susceptible to influence from bound modification. The subsequent disproportionate influence of these reactions (photon input in the case of autotrophically-growing organisms) on flux distributions may be viewed as problematic, because one portion of the solution space is more susceptible to reshaping while the rest of the network remains relatively unconstrained. In our results, however, METRADE* generally outperformed other methods when discriminating between conditions in PCoA and configuration choice seemed only to result in minimal differences in condition clustering, largely dispelling these concerns.

While not directly addressing the “top heaviness” of autotrophic systems, specifying bound constraints for key exchange reactions can lead to more robust solutions, with methods to quantify exchange reaction bounds having been proposed (Lin et al., 2025; Vijayakumar et al., 2020). Most previous validation studies, and case studies using continuous integration methods, focus on modelling cases where some experimental uptake rates are defined (even if there may be some methodological ambiguity in their determination), helping to shape final flux solutions (Lea-Smith et al., 2021; Joshi et al., 2020; Angione and Lió, 2015; Vijayakumar et al., 2018). A given integration method’s inferred predictive performance can vary greatly, however, depending on whether these uptake rates are manually set or left unconstrained (Lea-Smith et al., 2021; Blázquez et al., 2023). While E-flux2 and METRADE* have typically been implemented using specified uptake rates, it should also be noted that poorly substantiated input assumptions have the potential to bias output distributions - an important consideration since there is no uniform standard for defining such rates in autotrophic systems (Lin et al., 2025; Vijayakumar et al., 2018). In our analysis, we only specified the light input rate to facilitate a comparison between buffered and non-buffered configurations, leaving all other uptake rates undefined. In each case, light input flux was capped at an arbitrary baseline and subsequent growth rate predictions assessed in terms of their relative changes - a configuration similar to the “AC” (all possible carbon source) setup defined in previous validation studies (Joshi et al., 2020; Blázquez et al., 2023; Nielsen, 2003).

This approach was taken in the interest of maximising the number of viable conditions for analysis while also acknowledging that specific flux input rates are often not possible to obtain from previously published papers. For example, previous cyanobacterial metabolic modelling studies have calculated light available to cells using the culture surface area and dry cell weight per culture volume (Vijayakumar et al., 2020). The light availability value can be used to set specific photon exchange reaction bounds to reflect different lighting conditions. There is, however, no strict convention for reporting the values necessary for this light availability calculation among published work. For instance, flask type, height of the culture and dry cell weight measurements [which may require inference from a calibration curve at a suitable OD (Vijayakumar et al., 2018)] are often not included in transcriptomic publications. Even in cases where absolute or relative differences in light consumption by cells can be accurately estimated, the configuration of the starting bounds should remain an important consideration, because transcript-based photosystem regulation inference may still erroneously alter the entry of flux to the wider system, even if the extent of its influence is decreased.

### 3.1 METRADE* and E-flux2

Continuous methods, like E-flux2 and METRADE*, are sometimes preferred to discrete methods because they better reflect fine-grained expression changes while also avoiding categorisation of reaction expressions by somewhat arbitrary thresholds. This is partly supported by our findings, however, E-flux2 does not achieve the same level of success in condition separation as METRADE*. The ability of almost all METRADE* methods to outperform E-flux2 suggests there is a significant advantage in the use of the lazy-step mapping function with togglable scaling relative to E-flux2’s relative unit approach. The benefit of altering the “strength of mapping” appears largely reponsible for this difference, given that many of E-flux2’s model predictions achieve similar metrics to those of METRADE* with uniform scaling around 1 (“low strength”).

It is interesting that some methods which resulted in some of the tightest forming condition clusters in PCoA space, did not predict growth rates with the highest degree of accuracy. It appears, therefore, that clustering may be insufficient alone to determine the success of model contextualisation. This is probably because applying new constraints to a model has the capacity to separate conditions well in low-dimensionality space but risks overemphasising these differences beyond what would reasonably be expected in the true biological system. Growth rate accuracy can therefore serve to oppose clustering metrics by ensuring consistent relative differences between different time points in a manner which reflects those observed experimentally. We propose that successful model predictions should perform well across both metrics to ensure biologically feasible predictions. With this in mind, the best performing implementations tested (which are largely unaffected by configuration) are METRADE* with uniform scaling at gamma = 10, and variance-based scaling with maximum importance at 10,000 (Figure 4).

### 3.2 iMAT

Since iMAT is solved as a MILP, it does not rely on maximising a biomass equation (or any pre-computed objective function) to yield flux distributions. This, in theory, allows for increased flexibility within the solution space, potentially permitting more extreme changes between conditions. E-flux2- and METRADE*-based methods have objectives more rigidly defined, relying more heavily on solution space reshaping to guide flux distributions. Interestingly, in our PCoA simulations, biomass objective-reliant integration methods performed particularly strongly for condition clustering, in contrast to the highly variable performance seen among iMAT predictions (Figure 3A). Shifts in iMAT performance could be explained by the flexibility of MILPs, however, this flexibility seems to come at the cost of input parameter robustness.

While 13C-MFA is undoubtedly the preferred method for capturing the behaviour of cellular systems, it has rarely been used to select thresholds for switch-based methods. This analysis should be informative as iMAT thresholding decisions dictate which reactions are considered up- and downregulated, which in turn shapes the objective for optimisation. If the resulting objective demands flux in a manner which matches the fluxes across central carbon metabolism, we assume it is appropriate for modelling in the cyanobacterial model. Although this process acts as the opposing analysis to iMAT PCoA clustering (Supplementary Figure S1), it heavily relies on the assumption that the results are applicable to transcriptomic data from stress conditions and mutants (all infered from a WT transcript profile). Such an assumption is not easily testable given the lack of MFA data for *Synechocystis*, and therefore any iMAT PCoA clustering results should be interpreted with caution. While some more unusual percentile combinations showed great promise when assessed in low dimensionality space (particularly 0.35, 0.15), iMAT’s inability to predict biomass rates limits the confidence with which we can assess the biological plausibility of solutions with an opposing metric (as implemented for METRADE* and E-flux2) in this analysis.

### 3.3 Considerations

As Machado and Herrgård (2014) point out, transcriptomic integration methods are often tailored to address specific research questions; not necessarily for the precise prediction of intracellular fluxes (Machado and Herrgård, 2014). For example, across systems biology, integrative metabolic modelling is frequently used for the qualitative assessment of system behaviour or for comparative analyses between conditions (Vijayakumar et al., 2020; Uzuner Odongo et al., 2025; Tang et al., 2025).

In our analyses with METRADE*, we noted implementations that produced the tightest condition-specific clusters in PCoA tended to underperform in predicting growth rates. These types of outcomes suggest that when analysis pipelines are developed to detect fluxomic patterns associated with metabolic reprogramming, implementations which result in more distinct condition-specific clusters may be more effective at identifying relevant signals.

Inferred growth rates from OD measurements served as useful continuous trait targets for context-specific models. OD data, however, only allows for the estimation of relative growth rates, since it is not possible to determine absolute cell numbers from existing data. We accounted for the non-linear relationship between absorbance and cell number using the derivations described in Section 2.7. Curve fitting was relied upon to interpolate growth rates at time points where RNA was harvested but OD not explicitly measured. Fitted curves were checked visually and using standard goodness-of-fit metrics ( $R^{2}$ and RMSE).

## 4 Conclusion

Overall, this study evaluated the impact of different transcriptomic integration methods, making use of existing data from CyanoExpress. The report serves as a starting point for benchmarking integration methods in cyanobacteria and proposes strategies for reliable intracellular flux predictions. We quantifed the success with which iMAT, E-flux2 and METRADE* are able to produce context-specific model predictions which cluster in low-dimensional space. We also derived and used time-series growth rate traces, alongside clustering, to determine optimal scaling parameters and strategies when applying METRADE* - the best performing method given appropriate parameter choices. We observe how there is a trade-off between predicting growth rates and condition-specific clustering in low-dimensional space and discuss how different implementations may be considered optimal depending on the use case. Based on our results, METRADE* is the best choice for transcriptomic integration in *Synechocystis* metabolic models for the prediction of flux distributions retaining condition-specific properties, and for predicting growth rates.

## Data Availability

The original contributions presented in the study are included in the article/Supplementary Material, further inquiries can be directed to the corresponding authors. Additional files are on github: https://github.com/ThomasPugsley/Evaluating_transcriptomic_integration.

## References

[B1] Agren R. Mardinoglu A. Asplund A. Kampf C. Uhlen M. Nielsen J. (2014). Identification of anticancer drugs for hepatocellular carcinoma through personalized genome-scale metabolic modeling. Mol. Systems Biology 10 (3), 721. 10.1002/msb.145122 24646661 PMC4017677

[B2] Angione C. Lió P. (2015). Predictive analytics of environmental adaptability in multi-omic network models. Sci. Reports 5 (1), 15147. 10.1038/srep15147 26482106 PMC4611489

[B3] Beurton-Aimar M. Beauvoit B. Monier A. Vallée F. Dieuaide-Noubhani M. Colombié S. (2011). Comparison between elementary flux modes analysis and 13c-metabolic fluxes measured in bacterial and plant cells. BMC Systems Biology 5, 1–13. 10.1186/1752-0509-5-95 21682932 PMC3148577

[B4] Bhadra-Lobo S. Kim M. K. Lun D. S. (2020). Assessment of transcriptomic constraint-based methods for central carbon flux inference. Plos One 15 (9), e0238689. 10.1371/journal.pone.0238689 32903284 PMC7480874

[B5] Blázquez B. San León D. Rojas A. Tortajada M. Nogales J. (2023). New insights on metabolic features of bacillus subtilis based on multistrain genome-scale metabolic modeling. Int. J. Mol. Sci. 24 (8), 7091. 10.3390/ijms24087091 37108252 PMC10138676

[B6] Bordbar A. Mo M. L. Nakayasu E. S. Schrimpe-Rutledge A. C. Kim Y.-Mo Metz T. O. (2012). Model-driven multi-omic data analysis elucidates metabolic immunomodulators of macrophage activation. Mol. Systems Biology 8 (1), 558. 10.1038/msb.2012.21 22735334 PMC3397418

[B7] Caroline C. Aaron B. Zucker J. Lun D. S. Weiner B. Farhat M. R. (2009). Interpreting expression data with metabolic flux models: predicting mycobacterium tuberculosis mycolic acid production. PLoS Computational Biology 5 (8), e1000489. 10.1371/journal.pcbi.1000489 19714220 PMC2726785

[B8] Covert M. W. Schilling C. H. Palsson B. (2001). Regulation of gene expression in flux balance models of metabolism. J. Theoretical Biology 213 (1), 73–88. 10.1006/jtbi.2001.2405 11708855

[B9] Gopalakrishnan S. Joshi C. J. Valderrama-Gómez M. Á. Icten E. Rolandi P. Johnson W. (2023). Guidelines for extracting biologically relevant context-specific metabolic models using gene expression data. Metab. Eng. 75, 181–191. 10.1016/j.ymben.2022.12.003 36566974 PMC10258867

[B10] Gower J. C. (1966). Some distance properties of latent root and vector methods used in multivariate analysis. Biometrika 53 (3-4), 325–338. 10.2307/2333639

[B11] Guo W. Sheng J. Feng X. (2015). 13c-metabolic flux analysis: an accurate approach to demystify microbial metabolism for biochemical production. Bioengineering 3 (1), 3. 10.3390/bioengineering3010003 28952565 PMC5597161

[B12] Gurobi Optimization, LLC (2024). Gurobi optimizer reference manual.

[B13] Hernandez-Prieto M. A. Futschik M. E. (2012). Cyanoexpress: a web database for exploration and visualisation of the integrated transcriptome of cyanobacterium synechocystis sp. pcc6803. Bioinformation 8 (13), 634–638. 10.6026/97320630008634 22829745 PMC3400984

[B14] Hernández-Prieto M. A. Schön V. Georg J. Barreira L. Varela J. Hess W. R. (2012). Iron deprivation in synechocystis: inference of pathways, non-coding rnas, and regulatory elements from comprehensive expression profiling. G3 Genes– Genomes– Genet. 2 (12), 1475–1495. 10.1534/g3.112.003863 23275872 PMC3516471

[B15] Houot L. Floutier M. Marteyn B. Michaut M. Picciocchi A. Legrain P. (2007). Cadmium triggers an integrated reprogramming of the metabolism of synechocystis pcc6803, under the control of the slr1738 regulator. BMC Genomics 8, 1–16. 10.1186/1471-2164-8-350 17910763 PMC2190772

[B16] Hunter J. D. (2007). Matplotlib: a 2d graphics environment. Comput. Sci. and Eng. 9 (3), 90–95. 10.1109/mcse.2007.55

[B17] Hyduke D. R. Lewis N. E. Palsson B. Ø. (2013). Analysis of omics data with genome-scale models of metabolism. Mol. Biosyst. 9 (2), 167–174. 10.1039/c2mb25453k 23247105 PMC3594511

[B18] Joshi C. J. Peebles C. A. M. Prasad A. (2017). Modeling and analysis of flux distribution and bioproduct formation in synechocystis sp. pcc 6803 using a new genome-scale metabolic reconstruction. Algal Research 27, 295–310. 10.1016/j.algal.2017.09.013

[B19] Joshi C. J. Schinn S.-M. Richelle A. Shamie I. O’Rourke E. J. Lewis N. E. (2020). Standep: capturing transcriptomic variability improves context-specific metabolic models. PLoS Computational Biology 16 (5), e1007764. 10.1371/journal.pcbi.1007764 32396573 PMC7244210

[B20] Kim M. K. Lane A. Kelley J. J. Lun D. S. (2016). E-flux2 and spot: validated methods for inferring intracellular metabolic flux distributions from transcriptomic data. PloS One 11 (6), e0157101. 10.1371/journal.pone.0157101 27327084 PMC4915706

[B21] Laurent H. Arreckx S. Pfau T. Mendoza S. N. Richelle A. Heinken A. (2019). Creation and analysis of biochemical constraint-based models using the cobra toolbox v. 3.0. Nat. Protocols 14 (3), 639–702. 10.1038/s41596-018-0098-2 30787451 PMC6635304

[B22] Lea-Smith D. J. Summerfield T. C. Ducat D. C. Lu X. McCormick A. J. Purton S. (2021). Exploring the growing role of Cyanobacteria in industrial biotechnology and sustainability. 10.3389/fmicb.2021.725128 PMC8313982 34326831

[B23] Lin D.-W. Zhang L. Zhang J. Chandrasekaran S. (2025). Inferring metabolic objectives and trade-offs in single cells during embryogenesis. Cell Syst. 16 (1), 101164. 10.1016/j.cels.2024.12.005 39778581 PMC11738665

[B24] Machado D. Herrgård M. J. (2014). Systematic evaluation of methods for integration of transcriptomic data into constraint-based models of metabolism. PLoS Comput. Biol. 10 (4), 2014. 10.1371/journal.pcbi.1003580 24762745 PMC3998872

[B25] Nielsen J. (2003). It is all about metabolicfluxes. J. Bacteriology 185 (24), 7031–7035. 10.1128/jb.185.24.7031-7035.2003 14645261 PMC296266

[B26] Prakash J. S. S. Krishna P. S. Sirisha K. Kanesaki Yu Suzuki I. Shivaji S. (2010). An rna helicase, crhr, regulates the low-temperature-inducible expression of heat-shock genes groes, groel1 and groel2 in synechocystis sp. pcc 6803. Microbiology 156 (2), 442–451. 10.1099/mic.0.031823-0 19926653

[B27] Richelle A. Joshi C. Lewis N. E. (2019). Assessing key decisions for transcriptomic data integration in biochemical networks. PLoS Computational Biology 15 (7), e1007185. 10.1371/journal.pcbi.1007185 31323017 PMC6668847

[B28] Rohatgi A. (2011). Webplotdigitizer.

[B29] Shen F. Boccuto L. Pauly R. Srikanth S. Chandrasekaran S. (2019a). Genome-scale network model of metabolism and histone acetylation reveals metabolic dependencies of histone deacetylase inhibitors. Genome Biology 20 (1), 49. 10.1186/s13059-019-1661-z 30823893 PMC6397465

[B30] Shen F. Cheek C. Chandrasekaran S. (2019b). Dynamic network modeling of stem cell metabolism. Comput. Stem Cell Biol. Methods Protoc. 1975, 305–320. 10.1007/978-1-4939-9224-9_14 31062316

[B31] Shlomi T. Cabili M. N. Herrgård M. J. Palsson B. Ø. Ruppin E. (2008). Network-based prediction of human tissue-specific metabolism. Nat. Biotechnology 26 (9), 1003–1010. 10.1038/nbt.1487 18711341

[B32] Singh A. K. Elvitigala T. Bhattacharyya-Pakrasi M. Aurora R. Ghosh B. Pakrasi H. B. (2008). Integration of carbon and nitrogen metabolism with energy production is crucial to light acclimation in the cyanobacterium synechocystis. Plant Physiol. 148 (1), 467–478. 10.1104/pp.108.123489 18599646 PMC2528105

[B33] Singh A. K. Bhattacharyya-Pakrasi M. Elvitigala T. Ghosh B. Aurora R. Pakrasi H. B. (2009). A systems-level analysis of the effects of light quality on the metabolism of a cyanobacterium. Plant Physiol. 151 (3), 1596–1608. 10.1104/pp.109.144824 19759342 PMC2773086

[B34] Smith R. Ventura D. Prince J. T. (2013). Novel algorithms and the benefits of comparative validation. Bioinformatics 29 (12), 1583–1585. 10.1093/bioinformatics/btt176 23589651

[B35] Tang W. Lin S. Deng Y. Guo G. Chen G. Tianwei H. (2025). Integrating transcriptomic data with metabolic model unravels the electron transfer mechanisms of methanosarcina barkeri. bioRxiv, 10, 100379. 10.1016/j.wroa.2025.100379

[B36] Uzuner Odongo D. İlgün A. Bozkurt F. B. Çakır T. (2025). A personalized metabolic modelling approach through integrated analysis of rna-seq-based genomic variants and gene expression levels in alzheimer’s disease. Commun. Biol. 8 (1), 502. 10.1038/s42003-025-07941-z 40148444 PMC11950204

[B37] van ‘t Hof M. Mohite O. S. Monk J. M. Weber T. Palsson B. O. Sommer M. O. A. (2022). High-quality genome-scale metabolic network reconstruction of probiotic bacterium Escherichia coli nissle 1917. BMC Bioinformatics 23 (1), 566. 10.1186/s12859-022-05108-9 36585633 PMC9801561

[B38] Vijayakumar S. Conway M. Lió P. Angione C. (2018). Seeing the wood for the trees: a forest of methods for optimization and omic-network integration in metabolic modelling. Briefings Bioinformatics 19 (6), 1218–1235. 10.1093/bib/bbx053 28575143

[B39] Vijayakumar S. Rahman P. K. S. M. Angione C. (2020). A hybrid flux balance analysis and machine learning pipeline elucidates metabolic adaptation in Cyanobacteria. Iscience 23 (12), 101818. 10.1016/j.isci.2020.101818 33354660 PMC7744713

[B40] Wiechert W. (2001). 13c metabolic flux analysis. Metab. Engineering 3 (3), 195–206. 10.1006/mben.2001.0187 11461141

[B41] Yasemi M. Jolicoeur M. (2021). Modelling cell metabolism: a review on constraint-based steady-state and kinetic approaches. Processes 9 (2), 322. 10.3390/pr9020322

[B42] You Le Berla B. He L. Pakrasi H. B. Tang Y. J. (2014). 13c-mfa delineates the photomixotrophic metabolism of synechocystis sp. pcc 6803 under light-and carbon-sufficient conditions. Biotechnol. Journal 9 (5), 684–692. 10.1002/biot.201300477 24659531

[B43] You Le He L. Tang Y. J. (2015). Photoheterotrophic fluxome in synechocystis sp. strain pcc 6803 and its implications for cyanobacterial bioenergetics. J. Bacteriol. 197 (5), 943–950. 10.1128/JB.02149-14 25535269 PMC4325091

[B44] Young J. D. Shastri A. A. Stephanopoulos G. Morgan J. A. (2011). Mapping photoautotrophic metabolism with isotopically nonstationary 13c flux analysis. Metab. Engineering 13 (6), 656–665. 10.1016/j.ymben.2011.08.002 21907300 PMC3210925

[B45] Zamboni N. Fendt S.-M. Rühl M. Sauer U. (2009). 13c-based metabolic flux analysis. Nat. Protocols 4 (6), 878–892. 10.1038/nprot.2009.58 19478804

[B46] Zhang Z. Pendse N. D. Phillips K. N. Cotner J. B. Khodursky A. (2008). Gene expression patterns of sulfur starvation in synechocystis sp. BMC Genomics 9, 1–14. 10.1186/1471-2164-9-344 18644144 PMC2491639

[B47] Zur H. Ruppin E. Shlomi T. (2010). iMAT: an integrative metabolic analysis tool. Bioinformatics 26 (24), 3140–3142. 10.1093/bioinformatics/btq602 21081510

