# Supplementary material for: Evaluating transcriptomic integration for cyanobacterial constraint-based metabolic modelling
Source: Front Bioinform. 2026 Feb 4;6:1715377. doi: 10.3389/fbinf.2026.1715377 (PMC12913417; doi:10.3389/fbinf.2026.1715377)
Supplement: Supplementary file 1 [file DataSheet1.pdf]

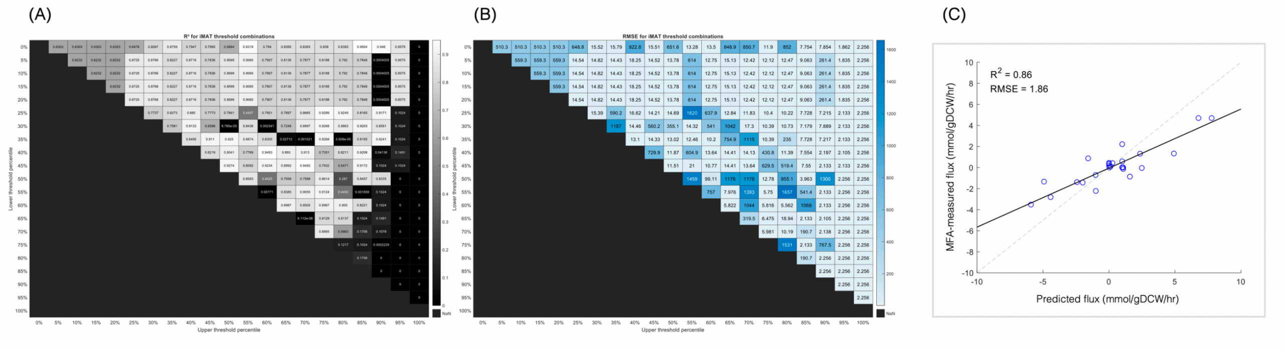

Supplementary Figure 1: R^2 (A) and RMSE (B) metrics comparing experimentally derived metabolic fluxes with those predicted when varying iMAT threshold combinations. An example of a predicted set of fluxes for a single threshold set (0.95, 0.00) is also shown (C).

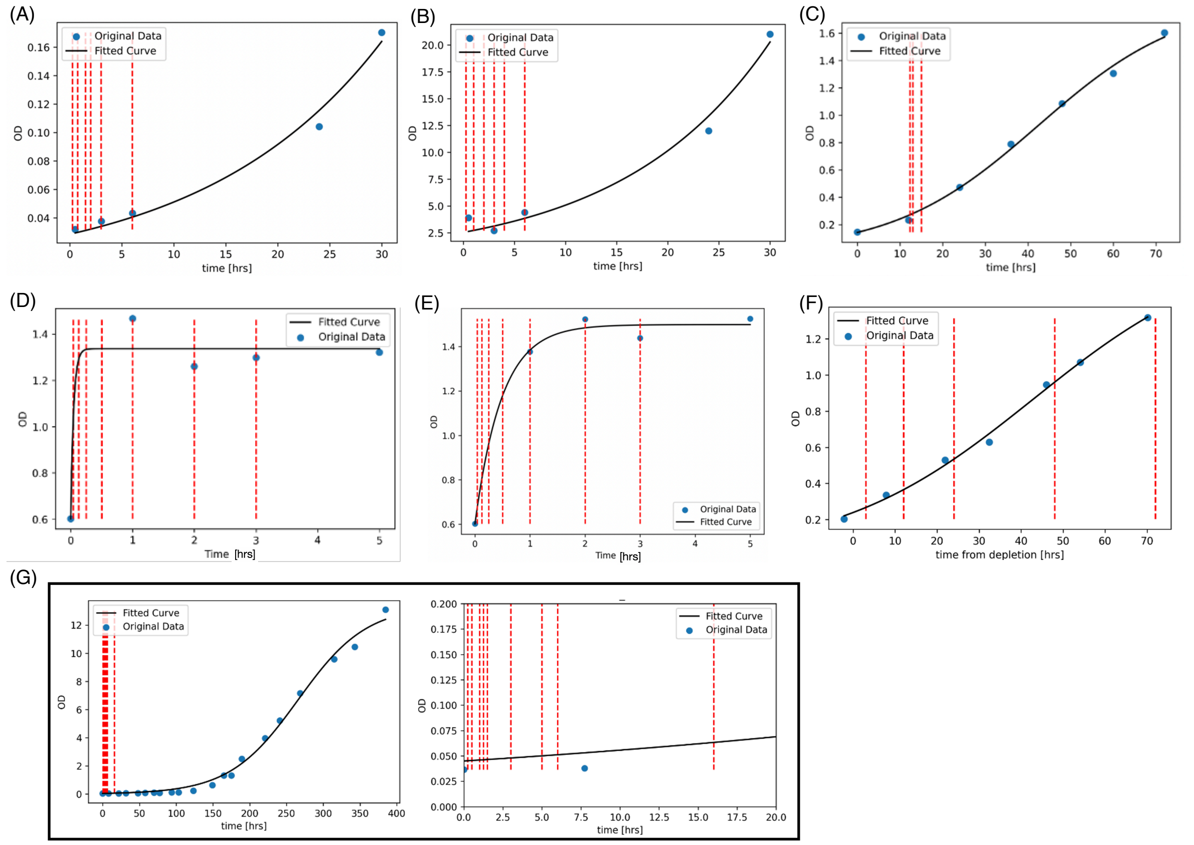

Supplementary Figure 2: Curves fitted to optical density datasets: ‘Blue light’ (A); ‘High light’ (B); ‘crhR’ (C); ‘S starvation (H)’ (D); ‘S starvation’ (E); Iron stress (F); Cd (G). Dashed lines represent time points at which relative growth rates were inferred.

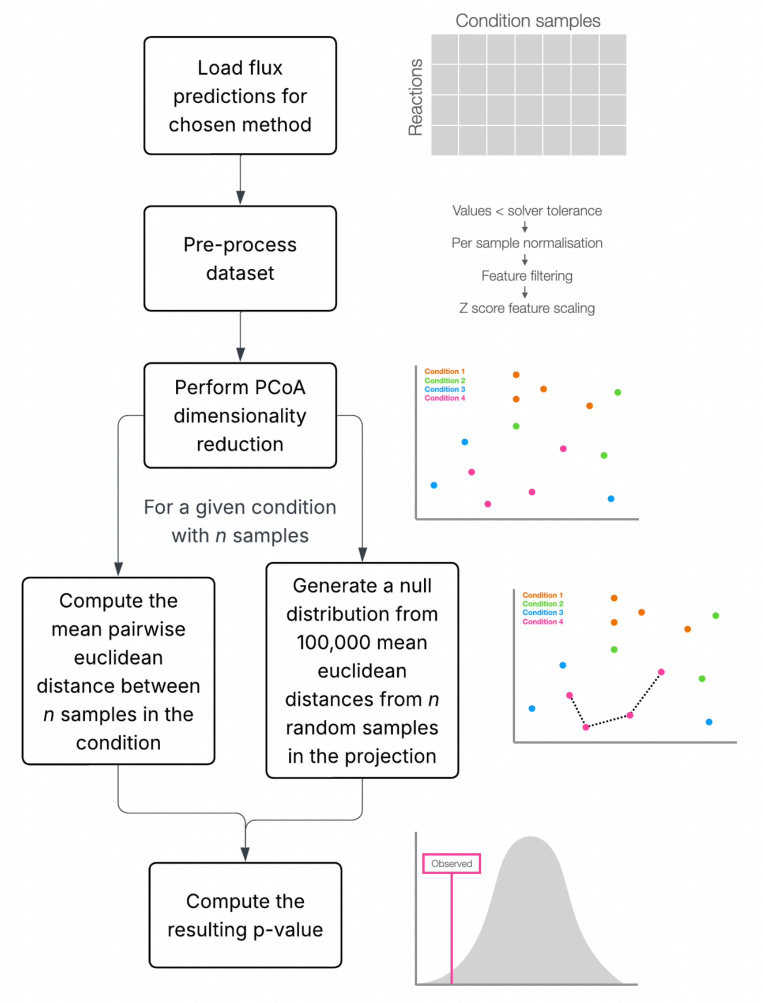

Supplementary Figure 3: Condition discrimination pipeline schematic
